# Survey on Colostrum Management by Dairy Farmers in the Netherlands

**DOI:** 10.3389/fvets.2021.656391

**Published:** 2021-04-06

**Authors:** Lisa Robbers, Hannes J. C. Bijkerk, Ad P. Koets, Lindert Benedictus, Mirjam Nielen, Ruurd Jorritsma

**Affiliations:** ^1^Population Health Sciences, Faculty of Veterinary Medicine, Utrecht University, Utrecht, Netherlands; ^2^Wageningen Bioveterinary Research, Lelystad, Netherlands

**Keywords:** survey, colostrum, calf feeding, colostrum management, dairy farm

## Abstract

Colostrum feeding is essential for the transfer of passive immunity and health of newborn calves. Information on current colostrum management practices to reduce calf morbidity and mortality is important but lacking for Dutch dairy herds. We therefore conducted a survey to investigate colostrum management strategies on Dutch dairy farms. The survey was specifically focused on the most recently born calf and was returned by 107 respondents (response rate of 13.4%). The mean amount of colostrum fed at first feeding was 2.9 liters. Overall, 79% of farmers provided the calf with at least 6 liters of colostrum in up to three feedings. The majority of respondents (84%) claimed to provide the calf with colostrum for the first time within 2 h post-partum. Using ordinal logistic regression and Wilcoxon rank sum test, we found no differences in time to first colostrum feeding or total amount of colostrum fed between bull calves and heifer calves, respectively. Ordinal logistic regression showed no significant differences in time to first colostrum feeding or time between calving and removing the calf from the dam between AMS and conventional milking herds. Two sample *T*-test comparing the total volume of colostrum showed no significant difference between AMS and conventional milking herds. Time of day at which a calf was born affected both volume fed at first colostrum feeding and time until first colostrum feeding. Calves born between 00.00 and 06.00 were significantly at risk of receiving the first colostrum later as compared to calves born at other times. Calves born in the evening received on average a lower amount of colostrum at first feeding. Survey results on colostrum management on most Dutch dairy farms are in agreement with the advice to feed as soon as possible after parturition and to provide at least 6 liters within 24 h of age. The current study points at time of calving as a potential risk factor for sub-optimal colostrum feeding. Further research is necessary to determine the consequences of this observation.

## Introduction

Calf health and mortality are important issues in the dairy industry. Calves are born agammaglobulinemic (1, 2) and rely for their first humoral specific immune protection on antibody transfer via (maternal) colostrum. Maternally derived antibodies from colostrum provide protection both locally in the gut and systemically after intestinal absorption (2, 3). This passive protection is crucial for neonates, as their immune system is fully developed, but lacks immunological memory. Insufficient uptake of maternal antibodies is termed Failed transfer of Passive Immunity (FPI) (2–4), and severely increases the risk of infections, disease and death. FPI used to be defined as a neonatal serum IgG concentration of 10 mg/mL or lower between 24 and 48 h of age (2–4) and for a long time the aim was to provide as much colostrum as needed to achieve a serum IgG concentration of ≥10 mg/mL. However, as new studies indicated that serum IgG concentration has a dose-response effect (5), this dichotomous approach was rather obsolete and a new and extended approach was presented by Lombard et al. (6) in which four categories of serum IgG concentrations are distinguished (excellent, good, fair and poor) which reflects the dose-response associations between serum IgG concentrations and calf morbidity and mortality risks. Multiple causes can lead to FPI and include the quality of freshly produced colostrum, storage and or treatments of colostrum and feeding methods of colostrum to the neonatal calf. Feeding colostrum of insufficient quality or quantity can lead to inadequate uptake of immunoglobulins. Feeding a calf too late hinders the intestinal absorption of maternal antibodies due to the process of gut closure (2, 4). As the antibody concentration decreases with every milking, and with a prolonged time between calving and milking, it is recommended to milk a cow completely and as quickly as possible after parturition (4, 7). In addition the supply of antibodies for the transfer of passive immunity, colostrum fulfills a range of other functions for the newborn calf. One of them is providing newborn calves with an adequate amount of energy to cover their relatively high energy requirements for thermoregulation (8). The nutritional value of colostrum is significantly higher than that of milk. The amount of fat is highly variable, but reported to be approximately 60% higher and the amount of casein is reported to be 90% higher compared to milk (9). Vitamins and minerals are present in a higher concentration as well (4). Moreover, colostrum contains high concentrations of growth factors, antimicrobial factors (10) and hormones (3).

The importance of adequate colostrum intake is undisputed and methods to ensure optimal colostrum feeding in practice are well-known. To cover the needs of the newborn calf, many advisors and farmers have adopted the “Three Q's” strategy: Quickly, Quantity, Quality. Additionally, a fourth Q, “sQueaky clean” is sometimes added (11), further termed “cleanliness.” This strategy highlights the importance of timely feeding of an adequate amount of good quality colostrum with minimal bacterial contamination. Still, calf morbidity and mortality are important issues. Improving on-farm colostrum management may present an opportunity to reduce calf morbidity and mortality. It has been suggested that farmers using an Automatic Milking System (AMS) have a different working routine than farmers using conventional milking systems (12). Also, there are indications that bull calves are treated differently from heifer calves with respect to the timing and volume of feeding colostrum (13). Knowledge on current colostrum management practices on dairy farms in the Netherlands is lacking. Therefore, the aim of this study was to investigate colostrum milking, storage, feeding and other colostrum related management methods currently applied in Dutch dairy farms. Additionally, we were interested whether dairy farms with automatic or conventional milking systems use different colostrum management methods and whether bull calves were treated differently from heifer calves. To this end, a survey was sent out to Dutch dairy farmers to inquire about colostrum management practices.

## Materials and Methods

### Colostrum Management Survey

The survey was developed by calf health experts at the Faculty of Veterinary Medicine at Utrecht University. Field experts and a communication consultant from the commercial dairy calf sector were involved to ensure content validity of the survey. Psychometric testing of the survey was not performed. Prior to distribution, a prototype survey was field tested with three dairy farmers to identify potential interpretation difficulties, and any unclear questions were adjusted.

We wanted to be able to identify a difference of 40 and 60% between AMS and conventional milking herds with 95% precision and a power of 80%. For this we needed a sample size of at least 100 respondents in each group. We expected a survey response of 25% and thus we sent out 400 invitations to participate in the survey to AMS farms and another 400 to farms with a conventional milking parlor. We could utilize a national database of milk equipment service organizations, which includes contact details of all Dutch dairy herds, following GDPR regulations. In total 800 Dutch dairy farmers were randomly selected and approached for participation.

The selected farmers were invited by an automated email in which they were asked to participate in the online questionnaire. The corresponding cover letter included information on the purpose of the research, a brief outline of the survey and the assurance that participation was completely voluntary and anonymous. Participants did not receive any remuneration. By clicking on a link provided in the email, respondents were directed to a survey software program (EvaSys Survey and Evaluation Software) where the survey started. To comply with national privacy regulations, the contact details of the farmers were not accessible for the researchers. Participants could provide their email address if they were interested in outcome of the study and potential additional in-depth interviews. Informed consent was given by clicking “yes” to the question if they would like to provide their email address, after which participants could provide their email address. It was assured that contact details would not be used for any other purposes. The online survey program was accessible between 11th of February 2019 and 25th of March 2019. A reminder to participate in the survey was sent out once at the 4th of March 2019.

The survey included a section on farm management, a section on technical information, a section on colostrum supply to the most recently born calf and a section on farmers' attitude and aspirations toward colostrum management. A full list of the questions in the survey can be found in Supplementary Table 1. Questions regarding farm management included whether the farm used an AMS, whether the milk produced was organic or not, and the age of the farm manager. Included technical information were the production results (305 days cumulative milk production (kg) and percentage fat and protein), the yearly percentage of calves that developed either respiratory disease or scours within 14 days of age, as well as the yearly percentage of calves that died withing 14 days of age. With respect to administration of colostrum, farmers were asked to specifically describe the details regarding colostrum management of the calf that was most recently born on their farm. By asking specifically about the most recently born calf we tried to minimize social desirability/response bias (i.e., describing best or generally applied practices rather than the actual on-farm colostrum management practices) and recall bias, given the assumed short period of time between calving and survey participation. In addition to questions related to the colostrum feeding methods performed with the most recently born calf, we inquired farmers' opinions on standard procedures with respect to colostrum supply. We asked opinions on several statements to which they could agree or disagree (Supplementary Table 2) or to which they could indicate a score on a seven point Likert scale (Supplementary Table 3).

### Data Handling and Statistical Analysis

For practical reasons and to conform to model assumptions of logistical models, levels within some dependent variables from the survey were grouped: Time to first colostrum feeding was grouped to (1) within 1 h, (2) within 2 h and (3) after 2 h. Farmers had the option to either report actual time of birth or, if they did not recall, to report the part of day in which the calf was born. All actual timepoints were converted to the part of day: morning (06:00–12:00), afternoon (12:00–18:00), evening (18:00–00:00) and night (00:00–06:00).

After data cleaning, R version 3.5.3 (Great Truth) and R studio version 1.3.959 were used for all descriptive and statistical analyses. The Shapiro-Wilks test was used to check for normality. When data was normally distributed, continuous data was compared using *t*-tests, if not, a Kruskal-Wallis combined with a Wilcoxon rank sum test was applied. Binary, categorical and ordered categorical data were assessed with logistic regression, multinomial logistic regression and ordinal logistic regression, respectively. For the ordinal logistic regression, the Brant test was used to assess parallel regression. The *p*-values of the logistical models were calculated using Analysis of Deviance (ANODE). Variables were dropped if they increased the AIC when included in a model. If dropping a variable from a model increased the AIC by <2.0, this variable was dropped in favor of a less complex model.

## Results

### Survey Response

Out of the 800 dairy farmers invited to participate in the survey (400 AMS, 400 conventional milking system) a total of 107 surveys responded, an overall survey response of 13.4%. A total of 62 farmers with an AMS system and 45 famers with a conventional milking system participated in the survey. Nine out of 107 surveys were incomplete (farm characteristics, day of calving and satisfaction with colostrum management). As the number of respondents was quite small and the missing values did not include data on colostrum management with the most recently born calf, we decided to include these incomplete surveys as much as possible to optimize the power of this study. We checked for biologically unrealistic values by histogram plots and checking for outliers. In some surveys some illogical values were reported: two farmers reported to have provided physiologically unlikely high amounts of colostrum with the third feeding (10 and 12 liters) and thus we reported these data as missing values. For a complete overview of the questions and number of corresponding answers, we refer to Supplementary Table 1.

### Population Description

**Figure 1:**
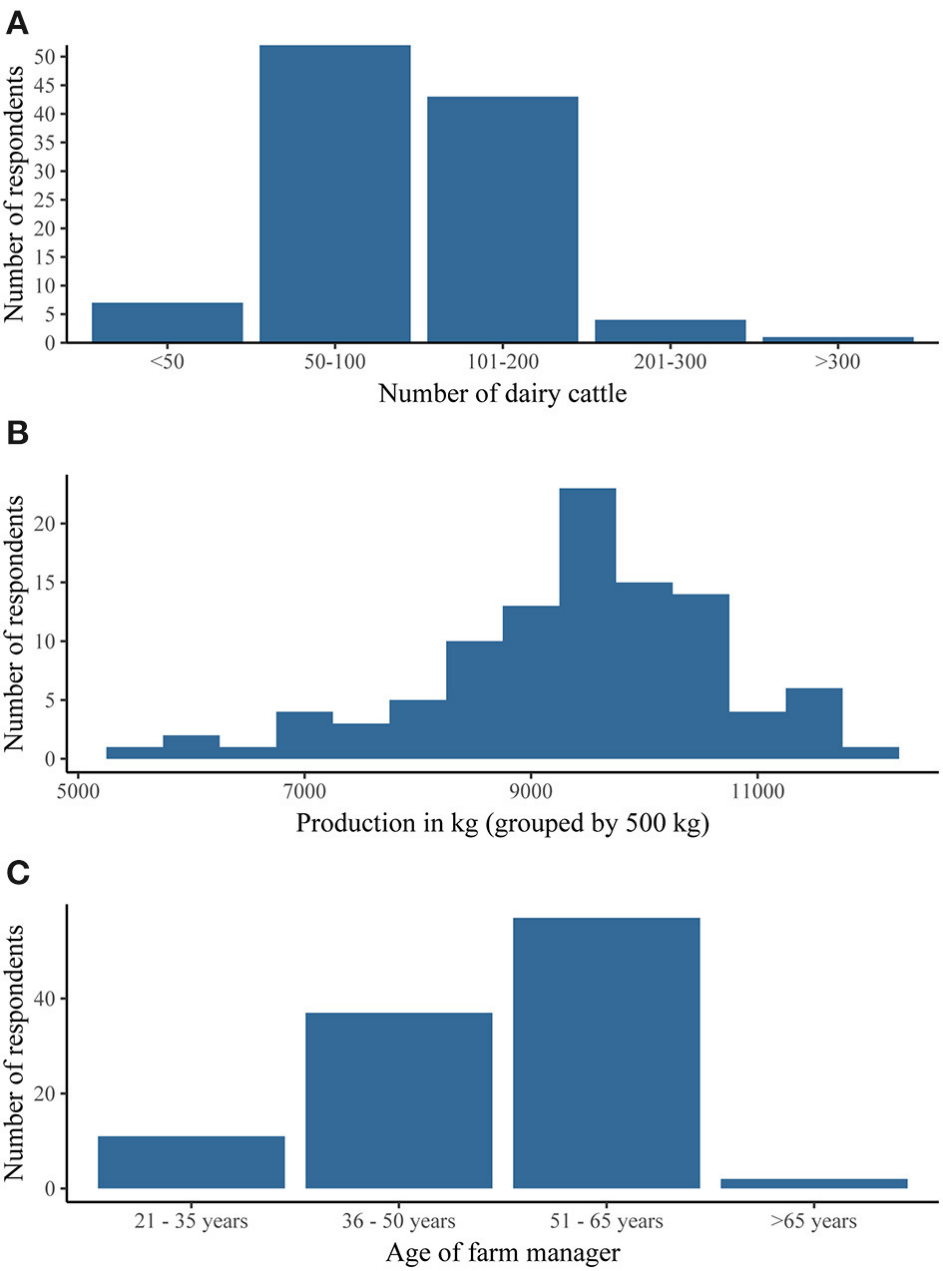
Distribution of respondents. The number of respondents (*n* = 107) grouped according to **(A)** herd size (*n* = 107), **(B)** 305 days milk production (*n* = 102) and **(C)** age of the farm manager (*n* = 107).

Of the 107 respondents, 7 (7%) produced organic milk, 98 (92%) used conventional farming methods, and 2 (2%) reported a different farming strategy. In Figure 1A the distribution of respondents' herd size is given. The 305 days cumulative milk production was grouped in categories of 500 kg and the number of respondents for each category is shown in Figure 1B. The mean 305 days production of the respondents was 9,388 kg (SD 1,282). The mean milk fat and milk protein percentage was 4.43% (SD 0.24) and 3.57% (± 0.12% SD), respectively. From the conventional milking farms, 44 (98%) out of 45 milked two times a day, and one milked three times per day. For the AMS farms the mean number of milkings per day was 2.8 (± 0.4 SD). The age distribution of the farm managers is displayed in Figure 1C. Distributions of calf calvings by part of day and by sex are given in Table 1.

**Table 1:** General calving statistics of the most recently born calf by time of calving and sex.

	**Morning (6:00–12:00)**	**Afternoon (12:00–18:00)**	**Evening (18:00–24:00)**	**Night (24:00–6:00)**	**Total**
Heifer	16	17	11	12	56
Bull	20	12	11	7	50
Total	36	29	22	19	106

### Amount of Colostrum Fed

**Figure 2:**
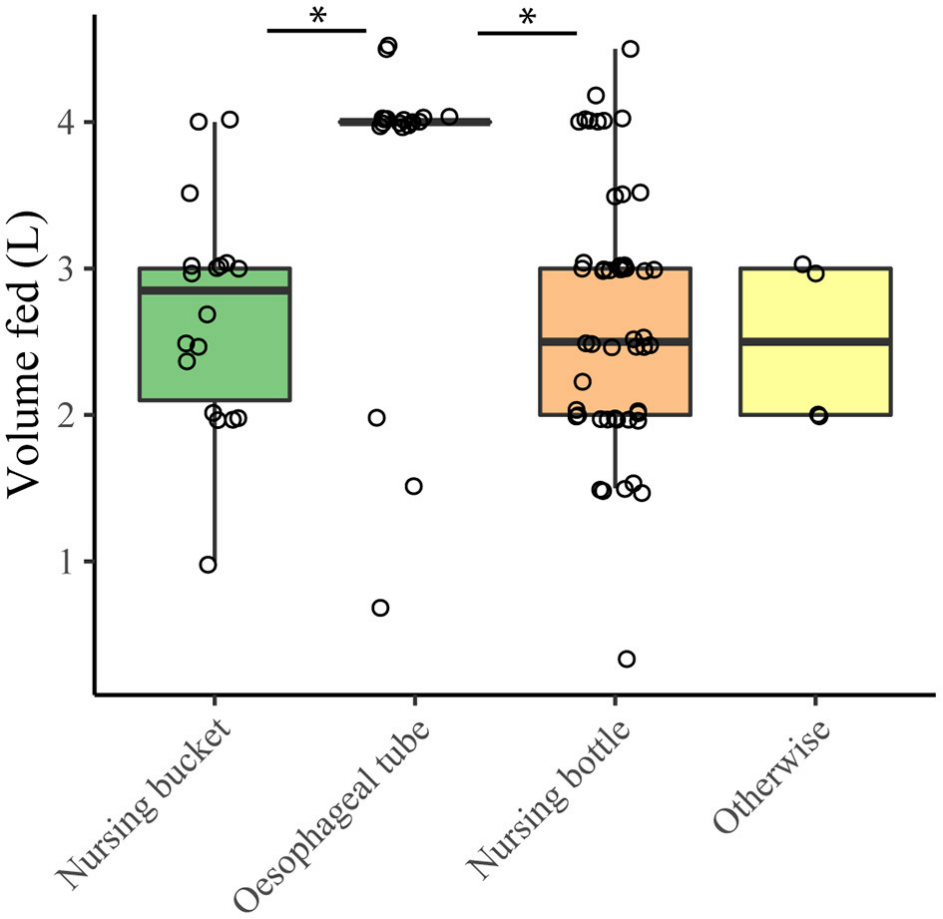
Volume of colostrum fed with the first colostrum feeding with different feeding methods (*n* = 89). *p < 0.05 (Wilcoxon rank sum test). The amount of colostrum fed with an esophageal tube was reported to be 4 liters 13 out of 18 times, resulting in the quartiles, minimum and maximum of the boxplot coinciding with the median.

**Figure 3:**
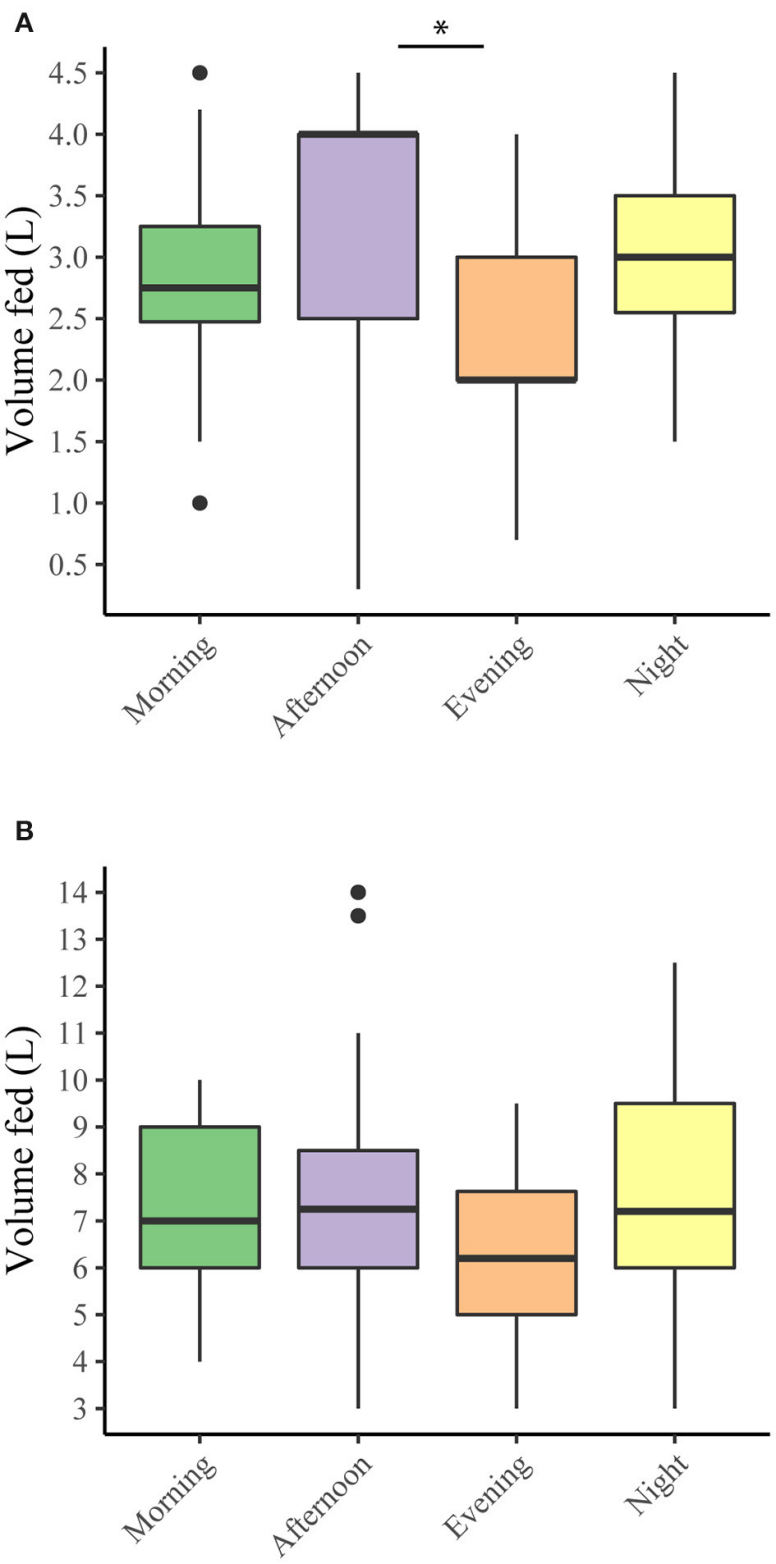
Volume of colostrum fed per calving time. **(A)**: The volume fed at first feeding (*n* = 89), **(B)**: The total volume of colostrum fed over three feedings (*n* = 84). *p < 0.05 (Wilcoxon rank sum test).

Of the 107 responders, 104 (97%) provided colostrum to the most recently born calf. The majority reported to feed the dams' own colostrum (Table 2). Of these 104, 15 farmers allowed the calf to suckle the dam for at least the first feeding, and another three farmers allowed the calf to suckle after the first feeding(s). Total volume ingested over three feeding moments could be determined for 84 respondents. The mean total amount of colostrum fed by 84 farmers known to feed all colostrum manually was 7.2 liters (SD 2.2). Out of these 84 farmers, 66 (79%) fed at least 6 liters of colostrum. An increase in the number of feedings was significantly (*p* < 0.01, Kruskal-Wallis) associated with a higher total colostrum supply. Also, method of feeding significantly (*p* < 0.01, Kruskal-Wallis) affected the volume provided at first feeding (Figure 2). Higher amounts were fed with an esophageal tube compared to feeding with a nursing bottle or a nursing bucket (*p* < 0.01, Wilcoxon rank sum). The amount of colostrum fed at first feeding was not the same for calves born at different times of the day (Figure 3A). Calves born during the afternoon received significantly more colostrum at first feeding than calves born during the evening (*p* < 0.001, Wilcoxon rank sum). However, total volume of feeding was not affected by the part of day in which a calf was born (*p* = 0.240, Kruskal-Wallis) (Figure 3B).

**Table 2:** Sources of colostrum fed to the most recently born calf.

	**Provided *n* (% of total)**	**From dam *n* (% of total)**	**From other cow *n* (% of total)**	**Mixed colostrum *n* (% of total)**
First feeding	104 (97.2%)	100 (96.2%)	3 (2.9%)	1 (1.0%)
Second feeding	97 (91.0%)	93 (95.9%)	3 (3.1%)	1 (1.0%)
Third feeding	90 (84.1%)	87 (96.7%)	0	3 (3.3%)

### Time Until First Feeding

**Figure 4:**
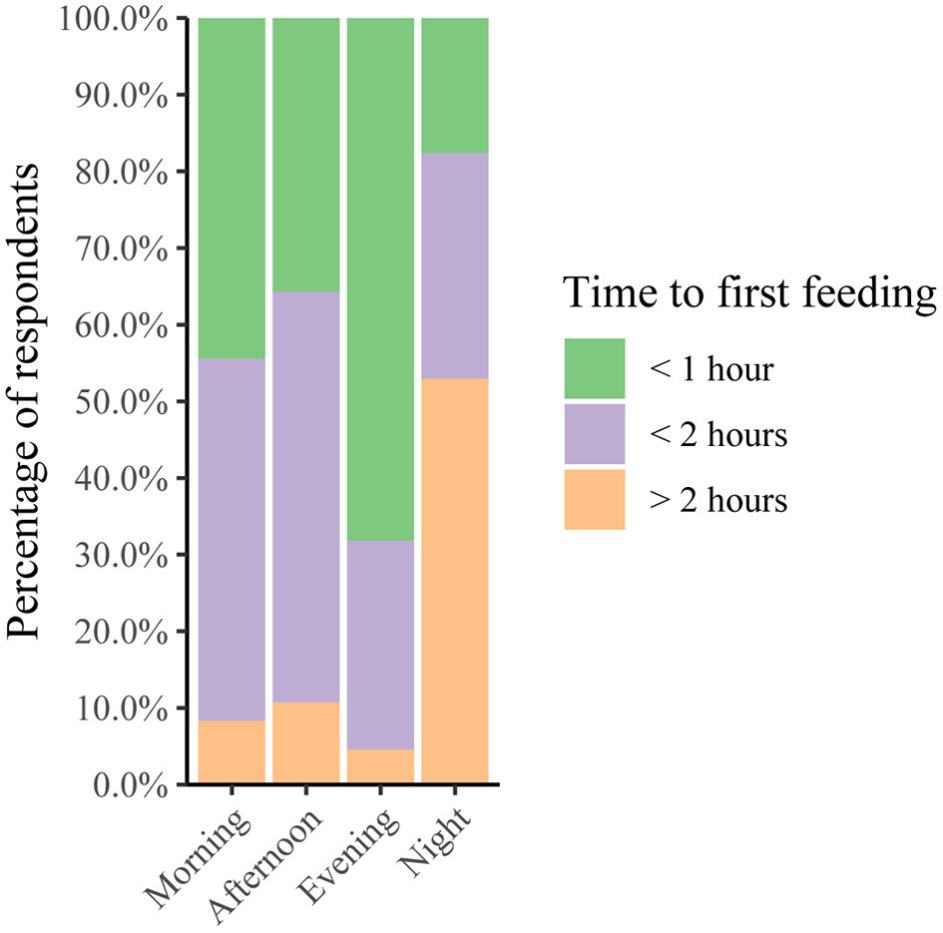
Time to first colostrum feeding with respect to time of calving of the most recently born calf (*n* = 103). Most calves born during morning, afternoon and evening receive first colostrum within 2 h after birth. At night however, half of the calves receives colostrum later than 2 h after birth.

Our results show that out of 104 calves receiving colostrum, 87 (84%) received the first colostrum within 2 h. Calves born at night had a significant higher risk to receive first colostrum later compared to calves born in the morning (OR = 8.21, CI 2.49–29.09), the afternoon (OR = 5.94, CI 1.76–21.46) and the evening (OR = 20.92, CI 5.35–90.48) (tested with ordinal logistic regression). Calves born in the evening tended to receive their first colostrum feeding sooner than calves born in the afternoon (Figure 4, Table 3).

**Table 3:** Estimated odds and 95% confidence interval (in brackets) of the odds to receive colostrum later between different times of birth.

	**Morning**	**Afternoon**	**Evening**
Afternoon	1.38 [0.54**–**3.53]		
Evening	0.39 [0.13**–**1.13]	0.28 [0.09**–**0.86]	
Night	8.21 [2.49**–**29.09]	5.94 [1.76**–**21.46]	20.92 [5.35**–**90.48]

As part of day of calving affected the time until and volume at first colostrum feeding, we examined whether this would affect method of feeding. We found that the multinomial logistic models with these dependent variables fitted our data better when part of day was dropped from the models. Thus, we found no influence of the part of day on feeding methods used (data not shown).

### Storage, Heating and Feeding Methods Used

All respondents together reported a total of 291 feedings. The majority of all colostrum feedings, 163 (56%), especially the first feeding, consisted of fresh colostrum given to the calf directly after milking (Table 4). Suckling the dam occurred in 24 (8%) of the total 291 feeding moments. Stored colostrum was almost exclusively used for 2nd and 3rd feeding. Colostrum was most frequently stored in the refrigerator, 46 out of 291 feeding moments (16%), followed by storage at room temperature, 29 out of 291 feeding moments (10%). Colostrum previously frozen and thawed was rarely used for feeding (three times, 1%). The other 9% of the total amount of feedings was stored differently. Most commonly used storage equipment were both sealed and open buckets (Table 5). A large proportion of the farmers, 29 out of 89 (33%), 37 out of 90 (41%) and 31 out of 88 (35%) reported to store the colostrum in open buckets, for feedings one, two and three, respectively. With respect to feeding equipment, colostrum was most frequently offered in a nursing bottle, particularly at first feeding (Table 6). At subsequent feedings, nursing buckets and normal buckets were increasingly used.

**Table 4:** Storage methods used (if applicable) prior to first, second and third feeding of colostrum to the most recently born calf.

	**Given directly after milking *n* (% of total)**	**Drank with dam*n* (% of total)**	**Stored frozen *n* (% of total)**	**Stored at room temperature*n* (% of total)**	**Stored refrigerated *n* (% of total)**	**Other*n* (% of total)**	**Total**
First feeding	85 (82%)	15 (14%)	2 (2%)	1 (1%)	0	1 (1%)	104
Second feeding	37 (38%)	7 (7%)	1 (1%)	13 (13%)	28 (29%)	11 (11%)	97
Third feeding	41 (46%)	2 (2%)	0	15 (17%)	18 (20%)	14 (16%)	90

**Table 5:** Storage containers used prior to first, second and third feeding of colostrum to the most recently born calf.

	**Sealed box *n* (% of total)**	**Sealed bucket*n* (% of total)**	**Open bucket *n* (% of total)**	**Nursing Bottle*n* (% of total)**	**Total**
First feeding	7 (8%)	29 (33%)	29 (33%)	24 (27%)	89
Second Feeding	12 (13%)	27 (30%)	37 (41%)	14 (16%)	90
Third Feeding	12 (14%)	33 (38%)	31 (35%)	12 (14%)	88

**Table 6:** Feeding method used at first, second and third colostrum feeding of the most recently born calf.

	**Nursing Bucket *n* (% of total)**	**Normal bucket*n* (% of total)**	**Nursing Bottle *n* (% of total)**	**Esophageal Tube*n* (% of total)**	**Other *n* (% of total)**	**Total**
First feeding	18 (20%)	0	49 (55%)	18 (20%)	4 (5%)	89
Second Feeding	39 (43%)	7 (8%)	42 (47%)	N/A^*^	2 (2%)	90
Third Feeding	51 (58%)	7 (8%)	29 (33%)	N/A^*^	1 (1%)	88

* *The option of using an esophageal feeding tube was not available for the 2nd and 3rd feeding*.

Four farmers out of the 104 farmer reporting to provide colostrum reported using colostrum from another source than the own dam. Two farmers fed for the first feeding frozen colostrum that was thawed using a hot water bath. One used colostrum from another dam, the other used pooled colostrum. One other farmer fed colostrum from another dam which was heated by a heat element. One farmer fed colostrum from one other dam, that was stored at room temperature. Out of the 90 farmers providing colostrum manually for the second time, 37 (41%) stated to have fed directly after milking the dam. Almost one third (31%) fed colostrum that had been stored in a refrigerator, while 14% fed colostrum that had been stored at room temperature. Only one farmer used frozen/thawed colostrum for feeding the second time. The remaining farmers ticked category “other” when asked about how they stored colostrum used at the second feeding. For the third feeding, 41 farmers (46%) supplied colostrum directly to the calf after milking the dam and two reported the calf drank with the dam. Storage in a refrigerator and at room temperature were reported by 18 (20%) and 15 (17%) of the farmers, respectively. None supplied colostrum that was previously frozen. The remaining respondents, 14, ticked “other” when asked about storage method.

### Comparing Colostrum Management Between Heifer and Bull Calves and Between Farms With Conventional and Automatic Milking Systems

**Figure 5:**
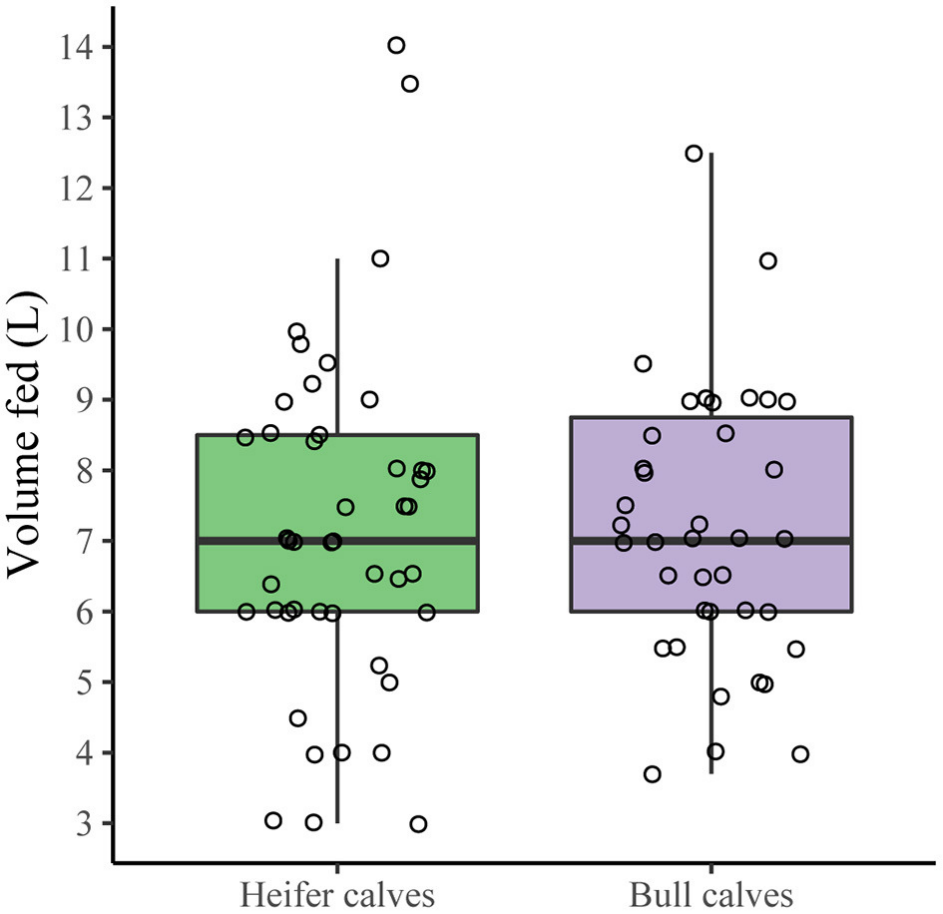
Total volume of colostrum fed to heifer calves and bull calves. Across all farms that provided colostrum manually for all three feedings (*n* = 84), no difference in total volume of colostrum fed was found between heifer and bull calves (*p* = 0.94, Wilcoxon rank sum test).

Analyzing the survey results on the most recently born calf, sex of the calf indeed did not affect the time to first colostrum feeding (ordinal logistic regression) (Table 7) or the volume of colostrum provided (*p* = 0.94, Wilcoxon rank sum test), as demonstrated in Figure 5. Also, no differences in colostrum management between AMS farms and farms with conventional milking systems were found in the current study for any of the dependent variables (time until first feeding of colostrum (ordinal logistic regression), total volume of colostrum fed (*p* = 0.41, two sample *T*-test), time between calving and removing calf from the dam (ordinal logistic regression), and the feeding method used for the first feeding (categorical logistic regression).

**Table 7:** Time to which heifer and bull calves receive their first colostrum.

	**Heifer calves**	**Bull calves**	**Total**
Within 1 h	25	19	44
Within 2 h	22	21	43
Later than 2 h	8	9	17
Total	55	48	104

### Farmers Opinions and Reported Standard Procedures With Respect to Colostrum Management

Farmers indicated that they did not differentiate colostrum feeding strategies between calves of different sexes, which was in agreement with the analyses of the most recently born calves in the survey. According to the questionnaire, 96 out of 103 (93%) responding farmers were satisfied with the way they provided colostrum to the most recently born calf. Out of all 107 respondents, 50 farmers defined colostrum as the first milking after calving (47%), while 44 (41%) farmers described it as milk from the first three milkings after calving and 11 (10%) described it as the first six milkings. The remaining respondents either defined colostrum as the first two milkings after calving (1%), or declared that it differs per dam (1%). As much as 34 (32%) reported that they routinely measure colostrum quality and 30 (28%) incidentally and the remaining 43 (40%) never measure colostrum quality. With respect to measuring colostrum quality, 45 (70%) farmers reported measuring colostrum with the use of a Brix refractometer, nine (14%) used a densimeter bobber and ten (16%) measured quality with a different method.

Almost half of the farmers, 46 out of 107 (43%), indicated that they never use an esophageal tube to feed calves. Thirty-eight respondents (36%) claimed to sometimes use esophageal tubes (10–40% of the calves), seven (7%) used it routinely (41–60% of the calves) and 4 (4%) frequently (61–90% of the calves). A total of 12 farmers (11%) claimed to always (more than 90% of the calves) use esophageal tubes for feeding colostrum.

Fifty out of the 107 (47%) farmers agreed with the statement that one of the most important reasons why calves sometimes cannot be fed the desired amount of colostrum in time, is that the first feeding of colostrum is delayed for a calf born at night compared to a calf born at other times of the day. However, only 19 (18%) farmers agreed with the statement that colostrum management is different between a calf born in the night compared to a calf born at other times of the day. Only one farmer agreed with the statement that colostrum management differs between bull- and heifer calves. The majority of farmers, 56 out of 107 (52%) stated that they acquired information on colostrum management from their veterinarian. Furthermore, 42 out of 107 farmers (39%) indicated they wanted more information on colostrum management from the veterinarian, 41 (38%) from the feed advisor, and 24 (22%) responded with a written answer: Eleven out of 25 answered that they were not in need of more information and nine farmers out of 25 indicated that they wanted more information regarding colostrum management from a specialist in calf rearing and one claimed to want more information from scientific research. The other three of the 25 written answers were not specific.

## Discussion

Proper management of colostrum feeding practices could potentially positively affect the uptake of immunoglobulins, thereby preventing FPI. In the search for optimal colostrum management, farmers and advisors have adopted the strategy: Quantity, Quickly, Quality and cleanliness. The aim of the survey was to investigate milking, storage, feeding and other colostrum management aspects as currently applied on Dutch dairy farms.

As results from a survey may not always reflect actual methods applied in practice, we specifically asked the farmers about the colostrum management methods they applied to the most recently born calf in order to reduce social desirability bias, and to minimize recall bias. Obviously, recall bias cannot be canceled out completely and therefore results should be interpreted with caution. For this type of survey we expected a response rate of ~25%, however the response rate of our study was relatively low and it is therefore difficult to extrapolate these results to the broader target group of dairy farmers in the Netherlands. We cannot exclude participation bias as it is possible that the subject of the survey may have attracted farmers with a special interest in calf rearing, while the online method may have refrained some farmers from responding.

To further determine the external validity of our study, we assessed the representativity of the respondents by comparing the results on farm characteristics to a national database. The most common reported herd size was 50 to 100 heads of cattle, which is in line with the average herd size of 99 heads of cattle in the Netherlands (14). The mean percentages for fat and protein reported in this survey were 4.47 and 3.57%, respectively, and are comparable to the means in the Netherlands, 4.38 and 3.59%, respectively (14). The mean 305 days milk production of the respondents was 9,388 kg, compared to 9,155 kg in the Netherlands (14). Considering the similarities, we think that with respect to farm characteristics (except for the high percentage AMS farms), our study population is representative of the situation in the Netherlands.

To identify a difference of 40 and 60% between AMS and conventional milking herds we aimed for a minimum of 100 participants in each group. Due to the low response rate, we only managed to achieve half the sample size we aimed for. Therefore, this sample size allowed us to only identify potential large effects (e.g., a difference of 30 and 60% between types of milking system). The precision of the study would have increased with a larger sample size, which would have allowed for smaller differences between groups to be detected.

One of our aims was to investigate whether colostrum management methods were different between dairy farms with a conventional and farms with an automatic milking system. Despite suggested differences in working conditions and time division between AMS farms and conventional milking system farms (12), no large differences in colostrum management strategies were found between farms using an AMS or a conventional milking system in survey. Additionally, we were interested whether colostrum management was different across farms between bull calves and heifer calves. Bull calves cannot replace dairy livestock in the herd and as such have less value to a dairy farmer. This might lead to farmers prioritizing colostrum feeding differently for heifers and bulls. Results from the survey indicate that with regard to colostrum management, across all farms both farmers' aims and actual practices applied to the most recently born calf did not differ between sexes. Similar to the results with respect to differences between AMS farms and farms with a conventional milking parlor, potential small effects could have remained undetected due to the limited sample size of the survey. Our results are different from results obtained by Shivley et al. (13), who reported that bull calves received first colostrum at a later timepoint compared to heifer calves. Also, bull calves were provided with a lower amount of colostrum over 24 h, compared to heifer calves. A possible explanation for these contradicting results could be that bull calves from participating herds left the operation at an average age of 7.6 days, while in the Netherlands bull calves are not allowed to be transported before 14 days of age. In this way, Dutch dairy farmers are responsible for the health status of their bull calves for a longer period.

Timely feeding of colostrum is essential, as the window for absorbing immunoglobulins by the gut is limited to 24 h post-partum and absorption is most optimal within 2 h of age (3, 15). With regards to Quickly feeding of colostrum, the majority of farmers (84%) reported feeding the first feeding of colostrum within 2 h or earlier, which coincides with commonly given advice to feed as quickly as possible (3). Our results are similar to those of Cummins et al. (16), who reported that the majority of the farmers in their study (84%) said to feed colostrum within 3 h after calving (16). The survey by Kehoe et al. (17), carried out on 55 dairy farms in Pennsylvania, US, revealed that only 44% of farmers fed colostrum within 2 h, 51% within 2–6 h, and 5% later than 5 h (17). With respect to timely feeding of colostrum, most progress could be made by paying more attention to calves born at night, as in our study, calves born at night received their colostrum significantly later than calves born at other times of the day. However, for practical reasons, this might be difficult to implement. Calves born in the evening receive colostrum the quickest, but also the least amount at first feeding. Possibly, the farmers are more flexible in their time during the evening, as other farm tasks are mostly performed in the morning and afternoon.

With regards to Quantity and frequency of colostrum feeding, we found that most farmers (84%) fed at least three servings of colostrum. In our study, 55% of the farmers that fed colostrum manually, supplied 3 liters or more with the first feeding, which is in line with the advice by Godden et al. (3). Adequate transfer of passive immunity is not only dependent on the volume, but is also affected by factors such as quality, contamination, and timing of the colostrum intake as well as birthweight. Obviously, farmers are not able to quantify these effects and this probably explains some of differences between the recommendations encountered in the literature. It is possible to maximize colostrum intake up to 3 liters with the use of an esophageal tube when calves do not voluntarily ingest at least 2 liters (15, 18). However, the farmers in our study appear to prefer repeated colostrum feedings to ensure sufficient uptake of IgG. The majority (79%) fed ≥6 liters in the three subsequent feedings we questioned, indicating small volumes at first feeding are compensated with subsequent feedings. These practices are in line with feeding at least 6 liters within 24 h as recommended (3, 19).

Quality of colostrum is usually expressed as the concentration of IgG in colostrum (3) and depends on many factors, such as such as breed (20), continuous milking (21), season of calving (22) and the time between calving and first milking (23, 24). Because of the large variation in colostrum quality, it can be useful to measure colostrum quality for example using Brix refractometry. Only about one third of our respondents reported to routinely measure colostrum quality. We recommend to pay more attention to measuring the quality of colostrum, because depending on the quality, it is possible to try to increase the volume to compensate for lower quality colostrum in order to achieve adequate transfer of passive immunity.

Another factor that attributes to quality of colostrum, is bacterial contamination. Storage of colostrum is known to affect total bacterial counts in colostrum (25, 26). While the majority of all feedings collectively was fed directly after milking the dam, many farmers in our survey reported to store colostrum in a refrigerator or at room temperature. Both Stewart et al. (25) and Cummins et al. (26) showed that storing colostrum at room temperature leads to a rapid increase in total bacterial counts within 24 h. Storing colostrum in a refrigerator delayed bacterial growth, however the effects were only temporary (25, 26). Not only are increased numbers of total bacteria associated with increased risk of disease, it may also lead to decreased absorption of colostral IgG by the newborn calf (27). We therefore recommend to store colostrum more often in a refrigerator instead of at room temperature to delay bacterial growth up until 24–48 h. For storing colostrum for a longer time, freezing and thawing of colostrum is recommended, as the immunoglobulin content is preserved while bacterial growth is diminished (28, 29).

Our survey provides insight in colostrum management practices by dairy farmers in the Netherlands. Our results indicate no systematic differences in colostrum management between dairy farms using an automatic or a conventional milking system. Across farms, heifer and bull calves were not treated differently. Furthermore, our results indicate that overall across the farms in our study, the common guidelines with respect to the “Four Q's” are mostly followed. The majority reported to feed Quickly, within 2 h of age). With regard to Quantity, approximately half of the respondents claimed to feed 3–4 liters colostrum at the first feeding and the majority, 79%, provides a total volume of ≥6 liters. More attention should be paid to measuring the Quality of colostrum, as this was not commonly measured in our survey and it helps determining the volume that should be fed. As for cleanliness, storing colostrum in a refrigerator rather than storing at room temperature should be considered to minimize bacterial growth. More attention should be paid to calves born at night, as they are at risk to receive colostrum later than calves born at other times of day, which affects the efficiency with which immunoglobulins are taken up by the calf.

## Data Availability Statement

The raw data supporting the conclusions of this article will upon request be made available by the authors, without undue reservation.

## Ethics Statement

Ethical review and approval was not required for the study on human participants in accordance with the local legislation and institutional requirements.

## Author Contributions

LR and RJ contributed to conception and design of the study. HB organized the database. HB, LB, and LR performed the statistical analysis. HB wrote the first draft of the manuscript. LR and HB wrote sections of the manuscript. All authors contributed to manuscript revision, read and approved the submitted version.

## Conflict of Interest

The authors declare that the research was conducted in the absence of any commercial or financial relationships that could be construed as a potential conflict of interest.
