# Supplementary material for: Survey on Colostrum Management by Dairy Farmers in the Netherlands
Source: Front Vet Sci. 2021 Apr 6;8:656391. doi: 10.3389/fvets.2021.656391 (PMC8056021; doi:10.3389/fvets.2021.656391)
Supplement: Supplementary file 1 [file Data_Sheet_1.docx]

Supplementary Material

# Supplementary Tables

## Supplementary table 1: Survey questions, number of respondents, and variable type

| **Question** | **Respondents** | **Variable type** |
| --- | --- | --- |
| Farm management type | 107 | Factor |
| Age of the farm manager | 107 | Factor |
| Number of cattle | 107 | Factor |
| Milking system | 107 | Factor |
| 305 days milk production (kg) | 104 | Continues variable |
| 305 days milk procution: fat % | 106 | Continues variable |
| 305 days milk production: protein % | 106 | Continues variable |
| BSK | 105 | Continues variable |
| Number of milkings (conventional milking system) | 45 | Factor |
| Number of milkings (automatic milking system) | 62 | Continue variable |
| Date of birth of the most recently born calf | 107 | Date |
| Is the time of calving known? | 106 | Factor |
| What is the time of calving | 87 | Time |
| What part of day did calving take place | 19 | Factor |
| What was the sex of the calf? | 107 | Factor |
| Aim to keep the heifer calf? | 56 | Factor |
| Course of parturition | 107 | Factor |
| What was the drylenght period of the corresponding dam? | 107 | Factor |
| Which lactation stage does the corresponding dam enter? | 107 | Factor |
| When was the calf removed from the dam? | 107 | Factor |
| Which date was the calf removed from the dam? | 31 | Date |
| Is the time known when the calf was removed from the dam? | 31 | Factor |
| What time was the calf removed from the dam? | 25 | Time |
| Which part of day was the calf removed from the dam? | 6 | Factor |
| When was the dam milked first after calving? | 107 | Factor |
| In what way was the dam milked? | 107 | Factor |
| Was the dam vaccinated against calf scours? | 107 | Factor |
| Did you supply the calf with colostrum? | 107 | Factor |
| What is the date when the calf received colostrum for the first time? | 104 | Factor |
| When did the calf receive colostrum for the first time? (hours after birth) | 104 | Factor |
| What was the colostrum source for the first colostrum feeding? | 104 | Factor |
| Was the calf nursed by the dam? | 100 | Factor |
| Was the dam milked out completely? | 85 | Factor |
| What was the volume of colostrum milked? | 86 | Continues variable |
| From which milking was the first colostrum given to the calf? | 3 | Factor |
| What volume of colostrum was fed to the calf at the first feeding? | 89 | Continues variable |
| What was the reason not to provide colostrum from the dam? | 4 | Factor |
| How did you store the colostrum that was given to calf? | 4 | Factor |
| What method did you use to thaw the colostrum? | 2 | Factor |
| Did you warm the colostrum before feeding to the calf? | 2 | Factor |
| What method did you use to warm the colostrum? | 3 | Factor |
| Did you check the temperature of the colostrum before feeding to the calf? | 89 | Factor |
| What was the temperature of the colostrum? | 10 | Continues variable |
| How was the colostrum stored before feeding it to the calf? | 89 | Factor |
| What method did you use to supply the calf with colostrum? | 89 | Factor |
| Did you save leftover colostrum? | 89 | Factor |
| After the first colostrum feeding, did you provide a second colostrum feeding? | 104 | Factor |
| What was the date of the second colostrum feeding? | 97 | Date |
| Do you know the time at which the second colostrum feeding was given? | 97 | Factor |
| At what time was the second colostrum feeding given? | 88 | Time |
| Which part of day was the second colostrum feeding given? | 9 | Factor |
| What was the colostrum source for the second colostrum feeding? | 97 | Factor |
| Was the calf nursed by the dam for the second colostrum feeding? | 94 | Factor |
| From which milking was the second colostrum given to the calf? | 90 | Factor |
| What volume of colostrum was fed to the calf at the second feeding? | 89 | Continues variable |
| How did you store the colostrum that was given to calf at second colostrum feeding? | 90 | Factor |
| What method did you use to thaw the colostrum for the second feeding? | 1 | Factor |
| Did you warm the colostrum before feeding to the calf at the second colostrum feeding? | 52 | Factor |
| What method did you use to warm the colostrum for the second feeding? | 49 | Factor |
| Did you check the temperature of the colostrum before feeding to the calf at the second colostrum feeding? | 90 | Factor |
| What was the temperature of the colostrum of the second feeding? | 11 | Continues variable |
| How was the colostrum stored before feeding it to the calf the second colostrum feeding? | 90 | Factor |
| What method did you use to supply the calf with colostrum for the second colostrum feeding? | 90 | Factor |
| Did you save leftover colostrum from the second feeding? | 90 | Factor |
| After the second colostrum feeding, did you provide a third colostrum feeding? | 97 | Factor |
| What was the date of the third colostrum feeding? | 90 | Factor |
| Do you know the time at which the third colostrum feeding was given? | 90 | Factor |
| At what time was the third colostrum feeding given? | 81 | Time |
| Which part of day was the third colostrum given to the calf? | 9 | Factor |
| What was the colostrum source for the third feeding? | 90 | Factor |
| Was the calf nursed by the dam for the third feeding? | 87 | Factor |
| From which milking was the third feeding given to the calf? | 85 | Factor |
| What volume of colostrum was fed to the calf at third feeding? | 88 | Continues variable |
| How did you store the colostrum that was given to the calf at third colostrum feeding? | 88 | Factor |
| What method did you use to thaw the colostrum for the third feeding? | 0 | Factor |
| Did you warm the colostrum before feeding to the calf at the third feeding? | 47 | Factor |
| What method did you use to warm the colostrum for the third feeding? | 39 | Factor |
| Did you check the temperature of the colostrum before feeding to the calf at the third colostrum feeding? | 88 | Factor |
| What was the temperature of the colostrum of the third feeding? | 11 | Continues variable |
| How was the colostrum stored before feeding it to the calf the third colostrum feeding? | 88 | Factor |
| What method did you use to supply the calf with colostrum for the third colostrum feeding? | 88 | Factor |
| Did you save leftover colostrum from the third feeding? | 88 | Factor |
| Are you satisfied with the way you have provided colostrum to the most recently born calf? | 103 | Factor |
| In your opinion, what is the definition of colostrum? | 107 | Factor |
| Additional explanation | 2 | Text |
| How do you recognize the quality of colostrum? | 105 | Text |
| Do you measure the quality of colostrum? | 107 | Factor |
| What method do you use to measure the quality of colostrum? | 64 | Factor |
| I measure the IgG concentration with Brix refractometry |  |  |
| I measure the IgG concentration with a colostrum densitometer |  |  |
| I measure the quality of colostrum in another way than stated here, namely: | 12 | Text |
| What do you aim for? Within how many hours do you aim to provide the first colostrum to heifer calves? | 107 | Continues variable |
| In how many times do you succeed in this aim? | 107 | Factor |
| What do you aim for? What volume of colostrum do you aim to provide within 12 hours of age to heifer calves? | 107 | Continues variable |
| In how many times do you succeed in this aim? | 107 | Factor |
| What do you aim for? within how many hours do you aim to provide the first colostrum to bull calves? | 107 | Continues variable |
| In how many times do you succeed in this aim? | 107 | Factor |
| What do you aim for? what volume of colostrum do you aim to provide within 12 hours of age to bull calves? | 107 | Continues variable |
| In how many times do you succeed in this aim? | 107 | Factor |
| In general, how often do you make use of esophageal feeding for colostrum supply to the calf? | 107 | Factor |
| What are the most important reasons why calves do not receive a desired volume of colostrum in a desired time? | 107 | Factor |
| If the calf is born at night, it will receive colostrum later than if it is born during the day. |  |  |
| If the calf drinks poorly, it will receive less colostrum |  |  |
| If the dam gives insufficient colostrum, the calf will receive less colostrum |  |  |
| Other reason, namely: | 29 | Text |
| From who did you obtain most information about the way of administering colostrum? | 107 | Factor |
| Animal feed advisor |  |  |
| Veterinarian |  |  |
| Professional magazines |  |  |
| Other dairy farmers |  |  |
| Predecessor (e.g. Parents or other relatives) |  |  |
| From who would you like to learn more about colostrum management? | 107 | Factor |
| Animal feed advisor |  |  |
| Veterinarian |  |  |
| Someone else, namely: | 24 | Factor |
| Colostrum management has a high priority on my farm | 107 | Factor |
| Optimal hygiene while milking and feeding colostrum has a high priority on my farm | 107 | Factor |
| In which way do you ensure optimal hygiene during collection and feeding of colostrum? | 107 | Text |
| It is important to be present at every parturition, even at night | 107 | Factor |
| Colostrum management on my farm is the same for all calves | 107 | Factor |
| Agree, every calf needs a sufficient amount of colostrum for a good start |  |  |
| Disagree, colostrum management is different for calvings during the day or during the night |  |  |
| Disagree, colostrum management is different and depends on sex of the calf |  |  |
| Agree, because… |  |  |
| Disagree, because… |  |  |
| Explanation agree/disagree colostrum management is the same for all calves | 69 | Text |
| I am actively working to optimize colostrum management | 107 | Factor |
| Explanation actively working to optimize colostrum management | 69 | Text |
| Do you know the KalfOK score of your farm? | 104 | Factor |
| What is the KalfOK score on your farm? | 56 | Continues variable |
| Over the past 12 months, what percentage of calves until 8 weeks of age has developed diahrea and/or respiratory infections? | 107 | Factor |
| Over the past 12 months, what was the percentage of calf mortality on your farm? (percentage stilborn or born within 14 days of age) | 106 | Factor |

## Supplementary table 2: Agreement of participating farmers with different statements on colostrum management

| Regarding colostrum feeding | Agreed | Disagreed |
| --- | --- | --- |
| If the calf is born at night, it will receive colostrum later than if it is born during the day. | 50 (47%) | 57 (53%) |
| If the calf drinks poorly, it will receive less colostrum | 42 (39%) | 65 (61%) |
| If the dam gives insufficient colostrum, the calf will receive less colostrum | 33 (31%) | 74 (69%) |
|  |  |  |
| Regarding whom gives information on colostrum management | **Agreed** | **Disagreed** |
| Animal Feed advisor | 42 (39%) | 65 (61%) |
| Veterinarian | 57 (52%) | 50 (48%) |
| Professional magazines | 36 (34%) | 71 (66%) |
| Other dairy farmers | 17 (16%) | 90 (84%) |
| Predecessor (e.g. Parents or other relatives) | 35 (33%) | 72 (67%) |
|  |  |  |
| Colostrum management is the same for every calf on my farm | **Agreed** | **Disagreed** |
| Agree, because every calf needs enough colostrum for a good start | 86 (81%) | 21 (19%) |
| Disagree, because management differs for day and night calvings. | 18 (17%) | 89 (83%) |
| Disagree, because management differs for bull- and heifer calves. | 1 (1%) | 106 (99%) |
| Agree, for different reasons | 4 (4%) | 103 (96%) |
| Disagree, for different reasons | 1 (1%) | 106 (99%) |

## Supplementary table 3: Farmers agreement on a Likert scale (1 – 7) on several topics regarding colostrum and calf management.

|  | Mean score | Standard deviation |
| --- | --- | --- |
| Colostrum Management has a high priority on my farm | 6,2 | 1,1 |
| Optimal hygiene while milking and feeding colostrum has a high priority on my farm | 6,0 | 0,9 |
| It is important to be present at every parturition, even at night | 4,5 | 1,8 |
| I am actively working to optimize colostrum management | 5,4 | 1,5 |

**
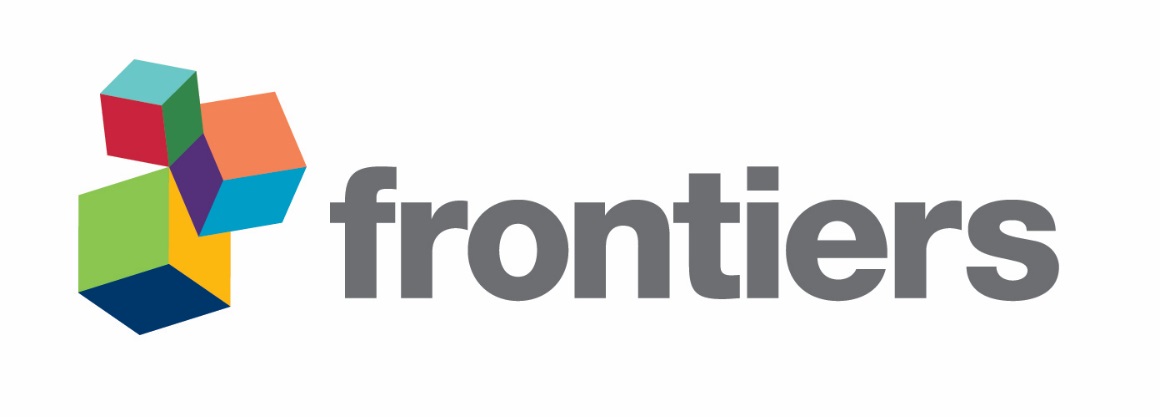
**
